# Mental pain and lifetime suicide attempts in early adolescence: a preliminary study

**DOI:** 10.1186/s13034-025-00883-8

**Published:** 2025-03-22

**Authors:** Jon García-Ormaza, Jeffrey V. Tabares, Ennio Ammendola, Alexander Muela

**Affiliations:** 1 Biobizkaia Health Research Institute, Bilbao, Bizkaia Spain; 2 Osakidetza Basque Health Service, Bizkaia Mental Health Network, Zamudio Hospital, Zamudio, Spain; 3 Medical School, University of the Basque Country UPV/EHU, Leioa, Bizkaia Spain; 4 Department of Psychiatry, The Ohio State University Wexner Medical Center, Columbus, OH USA; 5 Department of Clinical and Health Psychology and Research Methodology, University of the Basque Country UPV/EHU, Leioa, Spain; 6 Neuroscience Department, Biodonostia Health Research Institute, Gipuzkoa, Spain

**Keywords:** Suicide prevention, Youth suicide, Screening, Mental pain, Adolescents

## Abstract

The primary aim of the present study was to examine the association of four complementary measures—suicidal cognitions, entrapment, mental pain, and depression—with past suicide attempts in early adolescence. The sample consisted of 657 adolescents aged 12–15 years (*M* = 12.68, *SD* = ± 0.82; 49.16% female, 49.47% male and 1.22% non-binary), all enrolled in the first stage of secondary education (ISCED 2, first year) in schools in Spain. The study employed a three-phase analytical approach: (1) ROC/AUC analysis to evaluate the performance of each measure, (2) logistic regression models to assess their association with past suicide attempts, and (3) validating the first-phase classification results by accounting for the possibility of response imbalances on each of four measures. The results showed that high intensity of mental pain most strongly associated with past suicide attempts, exhibiting the highest sensitivity and reliability across models. Suicidal cognitions and entrapment also showed utility in assessing suicide risk, although their impact was less pronounced than mental pain. Depressive symptomatology showed limited utility distinguishing adolescents with a history of suicide attempts. These findings underscore the importance of incorporating mental pain and related constructs in community-based strategies for suicide prevention with early adolescence-aged children. By combining these factors, practitioners can gain a more comprehensive understanding of risk, facilitating early identification and intervention in adolescents at risk for suicide.

## Introduction

According to the latest available data [1], 47,346 deaths by suicide were recorded in the European Union in 2021, representing 0.9% of all deaths that year. This corresponds to an average rate of 10.2 deaths per 100,000 population, making suicide a significant public health issue in Europe.

In the pediatric population, particularly in the 15–19 age group, the average number of deaths by suicide between 2011 and 2021 was 4.47 per 100,000 [1]. Available data indicate that the highest rates are recorded in Eastern Europe, followed by Northern countries, while the lowest suicide rates are found in Southern Europe [1].

Although suicide is very rare in childhood, rates increase significantly from puberty onwards and continue to rise during early young adulthood [2]. A recent study [3] revealed a decrease in suicide rates in the 10–24 age group in most European countries in recent decades (1990–2020), although exceptions have been found where an upward trend has been identified in the United Kingdom, Ireland, Portugal, and Central as well as Eastern European countries. Outside Europe, in that age group, an increase in suicide rates has been observed in the United States (US) as well as in most of Central and Latin America and Oceania [3].

Suicidal ideation and behaviors are far more prevalent than death by suicide. In the United States, more than two million adolescents attempt suicide annually. The latest survey by the Centers for Disease Control and Prevention (CDC) [4] reported that 9.5% of high school students attempted suicide and 20.4% seriously considered it in the past year. Additionally, suicidal behavior has been observed to increase after the age of 12 years [5]. These statistics underscore the importance of implementing early detection and risk assessment systems for suicidal ideation and behavior, beginning in preadolescence at age 10 and continuing throughout adolescence [6, 7].

Suicidal thoughts and the transition to suicide attempts are distinct processes with different predictors and explanations [8]. The Interpersonal-Psychological Theory of Suicide (IPT) [9] was the first model to differentiate these processes. According to the IPT, individuals will die by suicide if they both desire to die (due to thwarted belongingness and perceived burdensomeness) and have the capability to act on these thoughts. Prevention strategies based on this theory emphasize modifying cognitive vulnerabilities and minimizing opportunities to act, including implementing personalized safety plans.

The Integrated Motivational-Volitional Model (IMVM) [10] expands on the IPT by focusing on the motivational phase, where feelings of defeat and entrapment foster suicidal intent, and the volitional phase, where intent transitions into action. Key factors in the volitional phase include access to lethal means, planning, and imitation behaviors.

In line with these distinctions, the Three-Step Theory (3ST) [11] posits that suicidal ideation arises from the combination of mental pain and hopelessness. The perception that life is painful and aversive drives this ideation, but hope for future improvement can deter individuals from contemplating suicide. Suicidal ideation intensifies when the combination of mental pain and hopelessness surpasses connectedness to life. Connectedness can refer to social bonds or a sense of purpose derived from projects, work, or personal interests. Transitioning from ideation to an attempt requires the capability to act on suicidal thoughts. According to the 3ST, this capability is influenced by predisposing factors (e.g., pain sensitivity, low fear of death), acquired experiences (e.g., child maltreatment, prior suicide attempts), and practical variables (e.g., access to lethal means). The 3ST suggests four key goals for suicide prevention: reducing mental pain, enhancing hope, strengthening connectedness, and reducing the capability for suicide. These assumptions have garnered significant empirical support in both adult [12] and youth populations [13].

In the US, the implementation of a suicide risk screening system in pediatric primary care has been promoted [6]. This measure is based on the notion that adolescents who die by suicide are more than twice as likely to have visited a primary care physician compared to a mental health specialist prior to their death [6]. Furthermore, most young people who die by suicide are known to visit health care professionals during the 6 months prior to their death by suicide [14], so actions have been implemented to highlight the importance of routine screening for suicidal behavior in adolescents, not only in primary care, but also in a variety of health care settings. These visits include emergency departments (ED), inpatient medical units and primary care clinics [15], health clinics [16] and inpatient medical-surgical units [17].

The implementation of this system has provided data supporting the need to establish universal screening for suicidal behavior. This approach identified suicide risk in approximately 3% of pediatric health care and ED visits, with a significantly higher prevalence of positive results in youth with psychiatric problems [15].

Many widely used instruments, such as the Ask Suicide-Screening Questions (ASQ) [18] or the Columbia Suicide Severity Rating Scale (C-SSRS) [19], primarily focus on suicidal ideation, a factor often associated with the early emergence of suicidal behavior. However, evidence suggests that suicidal ideation alone is an unreliable marker for predicting suicidal behavior [20–22]. Furthermore, in adults, it has been observed that more than one-third of those who attempt suicide or die by suicide deny suicidal ideation when screened [21]. It is also worth noting that recent studies indicate many people who attempt suicide do not experience suicidal ideation according to traditional definitions and measurements [23, 24]. These issues highlight the need to design screening and assessment tools tailored to the adolescent population based on diverse approaches to the nature of suicide risk [25]. Thus, instruments that focus exclusively on suicidal ideation need complementary assessments that capture additional dimensions of suicidal risk.

In this line, a key strategy that has gained support is the assessment of maladaptive beliefs and schemas, which can increase cognitive vulnerability to suicidal behavior, even in people without recent or previous suicidal thoughts or behaviors. In other words, complementary assessment seeks to identify a cognitive belief system that elevates suicide risk and explains the dynamic transition from chronic or baseline risk to acute risk [26]. This approach has the potential to improve the detection of patients who deny suicidal ideation during screening, as has already been demonstrated in studies with adult patients [23].

Internalizing symptoms are often included as a risk factor in suicide assessments, as they are associated with emotional problems such as depression and anxiety, which may increase vulnerability to suicidal ideation and behavior [27]. Health service professionals are very familiar with depression screening tools, such as the PHQ-A [28]. However, an important limitation has been identified: although depression is a relevant risk factor for suicide, not all adolescents at risk for suicide are depressed [27].

In a recent study of adolescent and young pediatric inpatients, aged 10–20 years [29], depression screening alone was found to miss nearly one-third of youths at risk for suicide. This suggests that a significant portion of at-risk adolescents presenting to health services may be missed if depression is used as the primary indicator to identify suicide risk. Therefore, it is recommended to complement depression screening with specific measures that assess both suicidal ideation and behavior [29].

Another variable that has shown a strong association with both the development and maintenance of psychopathology, and that acts as a direct driver of suicidal ideation and behavior, is the feeling of entrapment [30]. This concept refers to the perception of helplessness in the face of the impossibility of escaping situations of defeat or humiliation [31]. Recent findings [32] have shown that entrapment is a transdiagnostic factor and, due to its association with suicidal behavior, can be very useful for the detection and calibration of suicidal risk, providing greater explanatory variance than internalizing symptoms.

Finally, mental pain is now considered a risk indicator of suicidal ideation and behavior that has been integrated into various explanatory theories of suicidal behavior [11]. Shneidman [33] defined it as a state of aversion that encompasses a range of emotions including shame, guilt, humiliation, loneliness, fear, distress, apprehension, anguish, hopelessness, and rage. It is believed that when mental pain becomes unbearable, the person is more likely to move from suicidal ideation to action [11]. Despite mental pain not commonly being utilized as a complementary method of assessing suicidal behavior, a meta-analysis study [34] found an association between mental pain and suicidal behavior.

Based on the above, the main aim of the present study is to explore complementary variables associated with past suicide attempts in early adolescence, including mental pain, suicidal cognitions, entrapment, and depression. For this purpose, a cross-sectional study was conducted with a community sample of students aged 12–13 years. This approach aligns with the rationale for implementing early preventive strategies at a community level, addressing the need for universal interventions to effectively reduce suicide rates.

Despite advanced prevention strategies, youth suicide rates have risen significantly in countries like the US, where adolescent suicide deaths increased by 45.2% over the last decade [35]. These trends underscore the urgency of adopting universal, community-based strategies to reduce suicide rates effectively. This study also aligns with the “prevention paradox” [36], which suggests that most of the burden of a health problem (e.g., economic cost, mortality, and morbidity) stems from the population at low or moderate risk rather than from high-risk individuals. This paradox highlights why addressing the general population could prevent more cases of suicidal behavior or suicide deaths compared to focusing exclusively on high-risk clinical profiles [37]. By identifying factors associated with past suicide attempts, this study aims to contribute to the development of more comprehensive community-based risk assessment strategies, addressing the limitations of traditional approaches focused solely on suicidal ideation.

## Methods

### Participants

The sample consisted of 657 adolescents aged 12–15 years (*M* = 12.68, *SD* = ± 0.82; 49.16% female, 49.47% male, and 1.22% non-binary). Initially, 13 schools from various socioeconomic contexts in the Basque Country (northern Spain) were contacted to participate in the study. Of these, 12 schools agreed to collaborate. Letters were sent to the parents or legal guardians of 1047 students, inviting them to participate. Ultimately, 657 adolescents provided assent and parental or guardian consent, resulting in a participation rate of 62.75%. The inclusion criteria were adolescents enrolled in the first stage of secondary education (ISCED 2, first year). Adolescents with severe cognitive impairments or language barriers that prevented understanding of the questionnaire were excluded. Racial and ethnic composition was not explicitly recorded, as the study was conducted in schools in the north of Spain, where the majority of the population is of European descent.

### Procedure

We first contacted the schools to explain the purposes of the study, request their collaboration, and obtain their approval to participate. Subsequently, a letter was sent to the parents or legal guardians of the students, inviting them to participate in the study. The questionnaires were then administered at the schools. Informed consent was obtained from the parents or legal guardians of all participating minors, and assent was obtained from the students themselves.

Recent studies have shown that asking about suicide does not increase suicide risk [29]. Nonetheless, all necessary measures were taken during and after the completion of the instruments to ensure the emotional well-being of the participants. In cases where current suicidal thoughts with a plan, intent, or recent suicidal behavior (within the last month) were detected during the assessment, the parents or legal guardians of the participant were contacted to ensure that a responsible adult was aware of the risk and could help keep the participant safe. All safety procedures were communicated to both the participants and their parents or legal guardians through multiple channels. This information was included in the consent and assent forms, explained during the informational meeting prior to enrollment in the study, and reiterated before the completion of the assessment instruments.

The study was approved by the Ethics Committee for Research on Human Beings of the University of the Basque Country (approval code: act 004/2024).

### Measures

#### Lifetime suicidal ideation and suicidal behavior

Lifetime suicidal ideation and suicidal behavior was assessed with the Self-Injurious Thoughts and Behaviors Interview-Revised (SITBI-R) [38]. For the present study, the question “Have you ever tried to kill yourself? In other words, have you ever purposefully hurt yourself with some intent to die?” was used to determine whether the adolescents had made a suicide attempt. Responses were self-reported and recorded as “Yes” or “No”. Previous research supports the use of the self-report format of the SITBI-R, demonstrating that it has reliability and validity comparable to the interview-based format [29].

#### Suicidal cognitions

The Spanish Version of Suicide Cognitions Scale-Revised (Spanish SCS-R) [39] is a 16-item self-report questionnaire designed to measure a range of beliefs, attitudes, expectations, and perceptions associated with the emergence of suicidal thoughts and behaviors (e.g., “I don’t deserve to live another moment”). Items are scored on a five-point Likert-type scale capturing respondent agreement with each statement on a scale ranging from 0 (*strongly disagree*) to 4 (*strongly agree*). Item responses are summed to provide an overall metric of the suicidal belief system, with higher scores indicating increased vulnerability to suicidal thoughts and behaviors. Internal consistency in the present sample was ω = 0.95.

#### Depression

The Patient Health Questionnaire-Adolescent Version (PHQ-A) [40] consists of nine items (e.g., “Feeling down, depressed, or hopeless”) that assess the presence of depression symptoms over the past two weeks. The items are scored on a four-point Likert scale, from 0 (*never*) to 3 (*almost every day*). PHQ-A responses are summed to create a total score with higher scores indicating more severe depression symptoms. Internal consistency in the present sample was ω = 0.88.

#### Mental pain

A Brief Measure of Unbearable Psychache (UP3) [41] consists of three items that assess mental pain (e.g., “I can’t take my pain anymore”) on a five-point scale ranging from 1 (*strongly disagree*) to 5 (*strongly agree*). Items are summed, with higher scores indicating more intense and frequent (i.e., less bearable) mental pain. Internal consistency in the present sample was ω = 0.94.

#### Entrapment

The Entrapment Scale Short-Form (E-SF) [42] consists of four items (e.g., “I feel trapped inside myself”) that assess experiences of unbearable thoughts and feelings as well as unbearable situations from which one cannot escape. Items are scored on a five-point Likert scale ranging from 0 (*not at all like me*) to 4 (*extremely like me*). Higher scores on the scale indicate a higher degree of feelings of entrapment. Internal consistency in the present sample was ω = 0.88.

### Data analysis

The outcome of interest for all analyses was respondent endorsement of suicide attempt with some intent to die drawn from the SITBI-R. Analyses assessed the extent that suicidal cognitions, depression, mental pain, and entrapment predicted the outcome. Analyses proceeded in three steps. First, we generated a receiver operating characteristic (ROC) curve and area under the curve (AUC) for each measure, assessed properties of each ROC/AUC, and conducted pair-wise comparisons with DeLong’s test [43] that identified the better-performing model. Second, we performed a series of logistic regression models to identify the extent that measures of interest (separately or altogether) predicted endorsement of suicide attempt. Third, we validated our findings from the first step by accounting for potential response pattern imbalances in each of our four measures. Imbalances commonly occur in real-world data where classifiable events do not appear in equal proportions (e.g., true positive, false positive). Imbalances in responses could introduce imprecision on ROC/AUC properties reported. As a generic example, a measure where most respondents report ‘low severity’ (85%) relative to ‘high severity’ (15%) would have ROC/AUC properties aligning with majority cases (at the expense of minimal representation to minority cases). These proposed validations represent care with data handling and interpretation of AUC properties from balanced data. Observed similarity of AUC properties from analytic phases 1 and 3 provide support for the consistency of reported findings. We took two approaches at balancing response data and validating original ROC/AUC estimates: oversampling and undersampling from the existing dataset. Oversampling involves random draws from the minority response class; we used the synthetic minority oversampling technique (SMOTE). Undersampling involves random omission of majority cases to rebalance response classes; we used random undersampling (RUS). SMOTE and RUS each generated data-balanced ROCs and AUCs that were compared against original estimates from the first step.

## Results

### ROCs/AUCs, properties, and comparisons

**Fig. 1:**
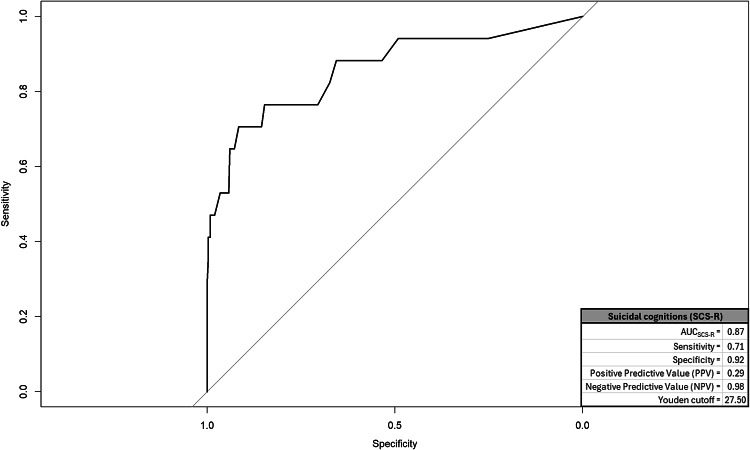
ROC and properties for suicidal cognitions

**Fig. 2:**
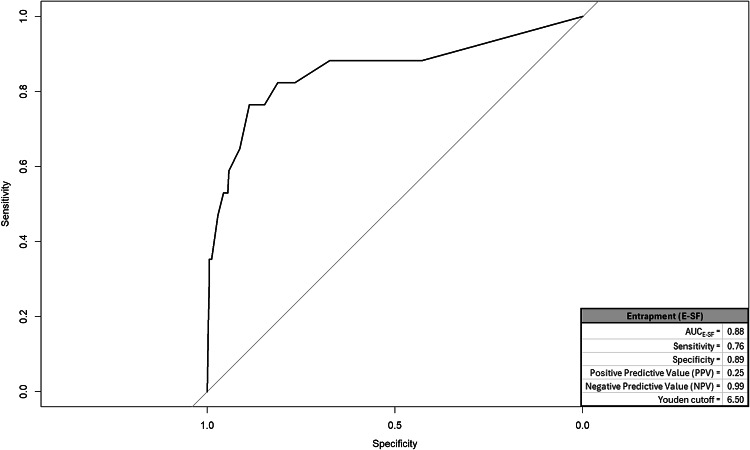
ROC and properties for entrapment

**Fig. 3:**
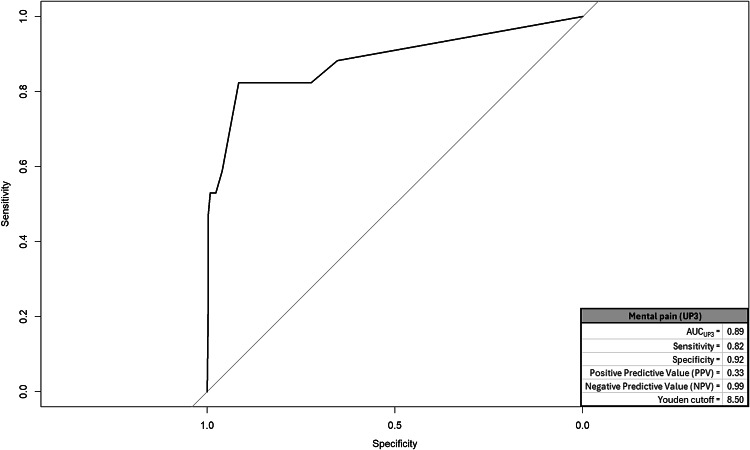
ROC and properties for mental pain

**Fig. 4:**
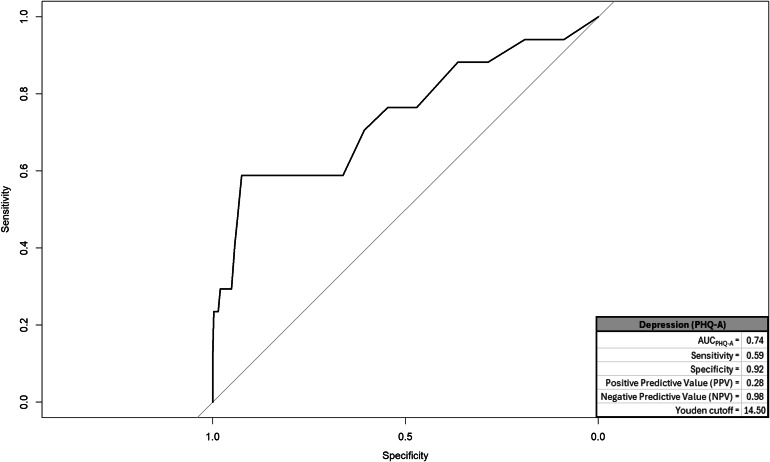
ROC and properties for depression

We created a ROC and corresponding AUC for suicidal cognitions, entrapment, mental pain, and depression; graphics for each appear in respective Figs. 1, 2, 3 and 4 and quantitative summaries of properties appear in Table 1. The values we observed at this first step indicated that mental pain was most capable in correctly distinguishing between true positive and negative endorsements for suicide attempt at varying sensitivity-specificity thresholds (AUC = 0.89). Mental pain had the highest rate of correct, true positive classifications among respondents endorsing suicide attempts (sensitivity = 0.82). Suicidal cognitions, mental pain, and depression each had high rates of correct true negative classifications among respondents who did not endorse suicide attempts (specificity = 0.92 for each). Utility of these measures to positively predict suicide attempt was generally low, but mental pain was the most predictive (0.33). All measures had high rates of correctly ruling out respondents who did not attempt suicide (0.98−0.99). Lastly, DeLong’s tests compared AUC values from each measure in a pairwise manner to identify the best-performing measure. A significant value on DeLong’s test would indicate the measure with higher AUC outperformed the competing measure. We had six pairwise comparisons, but all measures emerged as comparable (summary provided in Table 2).

**Table 1:** ROC/AUC properties by measure

	Estimates					
Measure	Area under the curve (AUC)	Sensitivity	Specificity	Positive predictive value (PPV)	Negative predictive value (NPV)	Youden cutoffs
Suicidal cognitions (SCS-R)	0.87	0.71	0.92	0.29	0.98	27.50
Entrapment (E-SF)	0.88	0.76	0.89	0.25	0.99	6.50
Mental pain (UP3)	0.89	0.82	0.92	0.33	0.99	8.50
Depression (PHQ-A)	0.74	0.59	0.92	0.28	0.98	14.50

The outcome measure was endorsement of suicide attempt with some intent to die. The Youden cutoff value for each measure represents a threshold score that balances sensitivity and specificity equally

*SCS-R* Spanish version of suicide cognitions scale-revised; *PHQ-A* patient health questionnaire-adolescent version; *UP3* the unbearable Psychache scale. Entrapment. *E-SF* entrapment scale short-form

**Table 2:** DeLong test values for pairwise comparisons between measures

DeLong tests			*z* Value	*p*-Value	95% Confidence interval	
Measure 1	vs.	Measure 2			Lower bound	Upper bound
Suicidal cognitions (SCS-R)	vs.	Entrapment (E-SF)	0.08	0.94	−0.08	0.08
Entrapment (E-SF)	vs.	Mental pain (UP3)	−1.31	0.19	−0.07	0.01
Mental pain (UP3)	vs.	Depression (PHQ-A)	1.66	0.10	−0.03	0.31
Depression (PHQ-A)	vs.	Suicidal cognitions (SCS-R)	1.43	0.15	−0.04	0.27
Suicidal cognitions (SCS-R)	vs.	Mental pain (UP3)	−0.57	0.57	−0.11	0.06
Entrapment (E-SF)	vs.	Depression (PHQ-A)	1.22	0.22	−0.07	0.29

The outcome measure was endorsement of suicide attempt with some intent to die

*SCS-R* Spanish version of suicide cognitions scale-revised; *PHQ-A* patient health questionnaire-adolescent version; *UP3* the unbearable psychache scale. Entrapment; *E-SF* entrapment scale short-form

### Logistic regression models

For the second analysis phase, we completed a series of logistic regression models to predict endorsement of suicide attempts while also controlling for the effects of age and sex: (1) separate models with each measure as a sole predictor and (2) a multivariable model with all measures included as predictors. For separate models, each measure emerged as a significant predictor of endorsement for suicide attempt. Mental pain emerged as the most impactful measure as single-unit increases were associated with a 65.74% log-odds *increase* of endorsing suicide attempt. Additionally, mental pain accounted for nearly 40.45% of variance associated with endorsing suicide attempt (i.e., Pseudo *R*^2^). Entrapment (40.79% log-odds increase, Pseudo *R*^2^ = 36.45%) and suicide cognitions (10.71% log-odds increase, Pseudo *R*^2^ = 35.65%) trailed mental pain for impact as second and third, respectively (Table 3 shows correlations among variables to identify potential multicollinearity while Table 4 summarizes findings for logistic regression models). For the multivariable logistic regression model, mental pain emerged as the only significant predictor accounting for a 53.07% log-odds increase toward endorsing suicide attempt. The multivariable model accounted for 50.82% of variance associated with endorsing suicide attempt. Variance inflation factor (VIF) values were generally low to suggest that relationships among variables would not affect resulting estimates and interpretations (Table 5 summarizes these findings).

**Table 3:** Zero-order correlations among proposed measures

	Suicidal cognitions (SCS-*R*)	Entrapment (E-SF)	Mental pain (UP3)	Depression (PHQ-A)
Suicidal cognitions (SCS-R)	–	0.79**	0.75**	0.54**
Entrapment (E-SF)		–	0.73**	0.52**
Mental pain (UP3)			–	0.50**
Depression (PHQ-A)				–

** Correlation is significant at the 0.01 level (two-tailed)

**Table 4:** Logistic regression estimates, predictor significance, and pseudo *R*^2^ (controlling for age and sex)

Measure as sole predictor	Logistic regression—coefficients							
	Estimate	Std. error	*z* Value	Pr(>|*z*|)	Odds ratio	95% Confidence interval		Pseudo *R*^2^
Suicidal cognitions (SCS-R)	0.10	0.01	6.93	0.00***	1.11	0.07	0.13	0.36
Entrapment (E-SF)	0.34	0.05	7.15	0.00***	1.41	0.25	0.44	0.36
Mental pain (UP3)	0.51	0.07	7.26	0.00***	1.66	0.38	0.65	0.40
Depression (PHQ-A)	0.17	0.04	4.61	0.00***	1.19	0.10	0.25	0.19
Significance codes:	0 ‘***’	0.001 ‘**’	0.01 ‘*’	0.05 ‘.’	0.1 ‘ ’	1		

The outcome measure was endorsement of suicide attempt with some intent to die. General model structure was: Outcome ~ Age + Sex + Predictor. Age and sex each appearing before the predictor of interest yielded predictor estimates that controlled for age and sex

*SCS-R* Spanish version of suicide cognitions scale-revised; *PHQ-A* patient health questionnaire-adolescent version; *UP3* the unbearable psychache scale. Entrapment; *E-SF* entrapment scale short-form

**Table 5:** Multivariable logistic regression estimates, predictor significance, and pseudo *R*^2^ (controlling for age and sex)

Measure as predictor	Logistic regression– Coefficients								
	Estimate	Std. error	z Value	Pr(>|z|)	Odds ratio	95% Confidence interval		Variance inflation factor (VIF)	Pseudo *R*^2^ (overall model)
Age (years)	0.41	0.54	0.77	0.44	1.51	−0.67	1.47	1.09	0.51
Sex	0.76	0.66	1.16	0.24	2.15	−0.50	2.11	1.09	
Suicidal cognitions (SCS-R)	0.05	0.03	1.40	0.16	1.05	−0.02	0.12	2.31	
Entrapment (E-SF)	0.02	0.11	0.21	0.84	1.02	−0.19	0.24	2.35	
Mental pain (UP3)	0.43	0.13	3.18	0.00**	1.53	0.17	0.70	1.92	
Depression (PHQ-A)	-0.01	0.06	-0.19	0.85	0.99	−0.13	0.11	1.31	
Significance codes:	0 ‘***’	0.001 ‘**’	0.01 ‘*’	0.05 ‘.’	0.1 ‘ ’	1			

The outcome measure was endorsement of suicide attempt with some intent to die. General model structure was: Outcome ~ Age + Sex + Predictor_1_… Predictor_N_. Age and sex each appearing before the predictors of interest yielded predictor estimates that controlled for age and sex

*SCS-R* Spanish version of suicide cognitions scale-revised; *PHQ-A* patient health questionnaire-adolescent version; *UP3* the unbearable psychache scale. Entrapment. *E-SF* entrapment scale short-form

### Validation

For the third and final step, we checked our original findings against revised analyses utilizing SMOTE and RUS that each accounted for the possibility of unbalanced data. Unfortunately, due to differences in model structures, we could not directly compare original, SMOTE, and RUS to identify the best-fitting model in a quantitative testing sense (e.g., similar to the DeLong’s test comparison described earlier). Specifically, the original estimates were drawn against a model requiring numeric structure on the outcome whereas both SMOTE and RUS utilized a factorial character structure. Given that disconnect preventing direct comparison between models, the findings we present as validation should be interpreted with caution (and as preliminary). Slight differences between model values may or may not represent significant differences. In the absence of a means to determine quantitative significance of model differences, we interpret the resulting numeric output at face value.

Revised analyses accounting for unbalanced data yielded results comparable to the original estimates. Mental pain was originally the most capable distinguishing between true positive and negative endorsements for suicide attempt (AUC_Original_ = 0.89), but maintained that status for SMOTE (AUC_SMOTE_ = 0.87) and RUS (AUC_RUS_ = 0.90). Additionally, mental pain maintained the highest sensitivity relative to other measures (Sensitivity_Original_ = 0.82, Sensitivity_SMOTE_ = 0.87, Sensitivity_RUS_ = 0.85). Lastly, mental pain maintained comparable specificity to the majority of other measures as was similarly observed for original estimates (Table 6 summarizes validation findings).

**Table 6:** Summary of validation values across original estimates, SMOTE, and RUS

Measure	Original estimates (from the first analytic step)			Synthetic minority oversampling technique (SMOTE)			Random undersampling (RUS)		
	AUC	Sensitivity	Specificity	AUC SMOTE	Sensitivity SMOTE	Specificity SMOTE	AUC RUS	Sensitivity RUS	Specificity RUS
Suicidal cognitions (SCS-R)	0.87	0.71	0.92	0.87	0.84	0.75	0.88	0.80	0.80
Entrapment (E-SF)	0.88	0.76	0.89	0.87	0.86	0.80	0.87	0.84	0.80
Mental pain (UP3)	0.89	0.82	0.92	0.87	0.87	0.80	0.90	0.85	0.85
Depression (PHQ-A)	0.74	0.59	0.92	0.72	0.78	0.55	0.77	0.72	0.65

The outcome measure was endorsement of suicide attempt with some intent to die

*SCS-R* Spanish version of suicide cognitions scale-revised; *PHQ-A* patient health questionnaire-adolescent version; *UP3* the unbearable psychache scale. Entrapment. *E-SF* entrapment scale short-form; *SMOTE* synthetic minority oversampling technique; *RUS* random undersampling

## Discussion

The aim of the present study was to identify alternative risk factors associated with past suicide attempts in early adolescence, including mental pain, suicidal cognitions, entrapment, and depression. These findings provide valuable insights that may inform future research into the potential utility of these factors for assessing imminent risk and developing improved strategies for suicide prevention.

The results revealed that high mental pain intensity, as measured through the UP3 [41], was the most strongly associated factor with past suicide attempts in early adolescence, compared with other assessed constructs such as suicidal cognitions, entrapment, and depression. Through ROC/AUC analyses and logistic regression models, high intensity of mental pain showed the highest sensitivity (82%) and positive predictive value (33%) in identifying suicide attempts. In the independent models, it was the strongest predictor, explaining 46% of the variance in attempts, while in the multivariable model it was the only significant predictor (49% of the variance). Likewise, data balancing techniques (SMOTE and RUS) confirmed the stability and reliability of mental pain, maintaining its high predictive capacity in different scenarios. Therefore, this result underscores the importance of considering mental pain as a relevant variable for estimating the risk or intensity of suicidal behavior in early adolescence. This finding aligns with theoretical frameworks such as the Three-Step Theory (3ST) and supports similar results observed in studies with older populations [34].

Although mental pain emerged as the factor most strongly associated with past suicide attempts, suicidal cognitions and entrapment also demonstrated relevance in understanding suicide risk. Both factors were significantly associated with past attempts in logistic regression models. In independent models, entrapment and suicidal cognitions contributed to increases in the log-odds of past suicide attempts (41% and 12%, respectively) and explained a considerable percentage of the variance (36% for entrapment and 40% for suicidal cognitions). These findings suggest that, while their individual impact does not exceed that of mental pain, suicidal cognitions and entrapment provide complementary insights into the psychological mechanisms underlying past suicide attempts. Therefore, these results also provide evidence in early adolescence supporting theories and studies conducted with older populations [23, 30], which highlight that the suicidal belief system and entrapment are relevant variables associated with suicidal behavior. These findings reinforce the notion that addressing these dimensions can offer a more comprehensive understanding of the factors contributing to suicide risk across different age groups.

One noteworthy finding from this study is that depression, as measured through the PHQ-A [40], exhibited a weaker association with past suicide attempts compared to other measures. While the PHQ-A demonstrated high specificity (0.92) and may be valuable for ruling out risk in adolescents who have not made suicide attempts, its association with actual attempts was limited. This suggests that, although the PHQ-A is a useful instrument for assessing an adolescent’s general emotional state, it may not fully capture the factors most closely associated with suicide attempts, even when specific items related to suicidal ideation are included. This finding is consistent with previous research [29], where depression is a broad risk factor, but not necessarily specific for predicting suicidal behaviors.

However, it is worth noting that other studies have found that item 9 of the PHQ-9, which measures the frequency of current suicidal ideation or self-harm thoughts, can identify adolescents at risk of suicide attempts and suicide [44]. Furthermore, recent research by Tsui and colleagues [45] specifies that the Patient Health Questionnaire–9 modified for teens (PHQ-9 M) is the instrument with the highest predictive capacity for suicide attempts. This enhanced predictive ability seems to stem from the four supplementary items that assess depression in the past year, functional impairment, severe suicidal ideation in the last month, and lifetime history of suicide attempts.

These results have important applications for community-based suicide assessment and prevention in early adolescence, as they provide a framework for identifying and prioritizing individuals at potential risk. While the ASQ [18] has been shown to be a robust measure for detecting suicidal ideation in adolescents [15], we believe that such measures should be complemented with other rapidly applicable screening tools, particularly since a significant percentage of individuals may choose not to disclose suicidal thoughts [21]. Given that mental pain emerged as the strongest associated factor, community practitioners can focus on this aspect during screening and/or evaluation. Combining the assessment of mental pain with suicidal cognitions and entrapment may offer a more comprehensive understanding of risk. These additional dimensions can help identify individuals who, even without experiencing acute mental pain, exhibit beliefs, attitudes, expectations, and perceptions associated with suicidal behaviors. This is particularly useful in community settings, where a multidimensional approach can enhance the detection of at-risk individuals and guide preventive interventions.

We consider this multidimensional strategy to be particularly useful in community settings, including schools, youth programs, and public health initiatives. These are key contexts where early identification of at-risk adolescents can be prioritized, especially since many adolescents may not explicitly disclose suicidal thoughts or behaviors. Furthermore, incorporating periodic assessments of complementary factors such as mental pain, suicidal cognitions, and entrapment in community programs could facilitate early preventive interventions in individuals whose risk status may fluctuate.

While this study focuses on a community sample, it is worth noting that these findings may have potential applications in healthcare settings, including emergency departments, where many adolescents present prior to a suicide attempt, often for somatic complaints [46]. Although not directly evaluated here, the combination of these indicators may reduce false negatives and improve the sensitivity of risk detection in such contexts. This could assist clinicians in making informed decisions about resource allocation and urgency of intervention based on a more reliable assessment of risk.

### Limitations

The results should be interpreted in the context of several limitations. First, suicide risk was assessed using self-report measures. Although self-report measures may yield further revelations about suicidal ideation and behavior [47], the results may not generalize to interviews or other methods of assessment. Second, the sample was drawn from a single geographic area and consisted of a community population, which may limit the generalizability of the findings. Future research should include clinical samples to explore whether the observed associations are consistent in high-risk populations. Additionally, analyses need to be replicated with data from other countries to generalize the results to different cultural and contextual settings. Third, this study focused specifically on suicide attempts as the outcome of interest. By focusing on attempts and not on suicidal ideation or non-suicidal self-injury, the study may have inadvertently omitted relevant factors in stages prior to the suicide attempt. Including both aspects in future research would allow for a more comprehensive analysis. Fourth, this study utilized a cross-sectional design. Future research with longitudinal data could test the predictive validity of the proposed screening and assessment of suicidal behavior presented in this paper. Despite these limitations, the results obtained provide valuable and useful information for screening and evaluating suicide risk in early adolescence, offering a robust basis for future research and the development of prevention strategies.

## Conclusion

This study identifies high intensity of mental pain as the factor most strongly associated with past suicide attempts in early adolescence, outperforming suicidal cognitions, entrapment, and depression. With high levels of sensitivity and specificity, mental pain emerged as the only significant factor in a multivariable model, while suicidal cognitions and entrapment also contributed valuable insights in individual models. These findings highlight the importance of incorporating multidimensional assessments in community-based strategies for suicide prevention. By considering mental pain alongside suicidal cognitions and entrapment, practitioners can gain a more comprehensive understanding of suicide risk, facilitating early identification and intervention. Although this study focuses on a community sample, its implications may extend to other settings, such as healthcare environments, where these constructs could enhance the detection of at-risk adolescents and reduce false negatives.

## Data Availability

Data available on request due to privacy/ethical restrictions.
